# Predicting Mortality in Pulmonary Embolism: A Machine Learning Approach with External Validation in COVID-19 Patients

**DOI:** 10.3390/medicina62020421

**Published:** 2026-02-23

**Authors:** Diana Alexandra Mîțu, Alexandru Cristian Cindrea, Alexandra Maria Borita, Adina Maria Marza, Corneluța Fira-Mladinescu, Madalin-Marius Margan, Alexandra Herlo, Alina Petrica, Gabriel-Aurel Rus, Daniel-Florin Lighezan, Flavia Zara, Ovidiu Alexandru Mederle

**Affiliations:** 1 Doctoral School, Faculty of General Medicine, “Victor Babes” University of Medicine and Pharmacy, 300041 Timisoara, Romania; diana-alexandra.mitu@umft.ro (D.A.M.); alexandru.cindrea@umft.ro (A.C.C.); 2 Department of Internal Medicine, Emergency Clinical Municipal Hospital, 300254 Timisoara, Romania; alexandraborita@gmail.com; 3 Emergency Department, Emergency Clinical Municipal Hospital, 300254 Timisoara, Romania; mederle.ovidiu@umft.ro; 4 Department of Surgery, “Victor Babes” University of Medicine and Pharmacy, 300041 Timisoara, Romania; alina.petrica@umft.ro; 5 Discipline of Hygiene, Department of Microbiology, Center for Studies in Preventive Medicine, “Victor Babes” University of Medicine and Pharmacy, 300041 Timisoara, Romania; 6 Discipline of Public Health, Department of Functional Sciences, “Victor Babes” University of Medicine and Pharmacy, 300041 Timisoara, Romania; margan.madalin@umft.ro; 7 Center for Translational Research and Systems Medicine, Faculty of Medicine, “Victor Babes” University of Medicine and Pharmacy, 300041 Timisoara, Romania; 8 Department of Infectious Diseases, “Victor Babes” University of Medicine and Pharmacy, Eftimie Murgu Square 2, 300041 Timisoara, Romania; alexandra.mocanu@umft.ro; 9 Emergency Department, “Pius Brinzeu” Emergency Clinical County Hospital, 300736 Timisoara, Romania; 10 Department of Medical Oncology, Emergency Clinical Municipal Hospital, 300254 Timisoara, Romania; gabrielrua8@gmail.com; 11 Department of Medical Semiology I, “Victor Babes” University of Medicine and Pharmacy, No. 2 Eftimie Murgu Square, 300041 Timisoara, Romania; dlighezan@umft.ro; 12 Advanced Cardiology and Hemostaseology Research Center, “Victor Babes” University of Medicine and Pharmacy, No. 2 Eftimie Murgu Square, 300041 Timisoara, Romania; 13 Department II of Microscopic Morphology, “Victor Babes” University of Medicine and Pharmacy, E. Murgu Square, No. 2, 300041 Timisoara, Romania; flavia.zara@umft.ro; 14 Angiogenesis Research Center, “Victor Babes” University of Medicine and Pharmacy, E. Murgu Square, No. 2, 300041 Timisoara, Romania

**Keywords:** pulmonary embolism, SARS-CoV-2, risk stratification, PESI, machine learning

## Abstract

*Background and Objectives*: Pulmonary embolism (PE) is a frequent thrombotic complication associated with SARS-CoV-2 infection and is linked to significant early mortality. Accurate early risk stratification in the emergency department (ED) remains challenging, and it is unclear how well commonly used PE prognostic tools perform in patients with concomitant COVID-19. *Materials and Methods*: We conducted a retrospective, single-centre study including 538 consecutive patients with acute PE and with or without confirmed SARS-CoV-2 infection admitted through the ED. Univariate analysis and machine learning models were employed to assess mortality risk. *Results*: In univariate analysis, mortality was strongly associated with sepsis (OR 11.68) and PESI class V (OR 5.56) and was also linked to higher neutrophil count (OR 1.19), platelet count (OR 1.12), and NT-proBNP (OR 1.20). In the non-COVID cohort, XGBoost and RF showed better discrimination than PESI class (AUC 0.864 and 0.834 vs. 0.725), while Support Vector Machines (SVM) was lower (AUC 0.740). On COVID-19 external validation, discrimination decreased: XGBoost AUC was 0.635, RF 0.614, PESI 0.584, and SVM showed no discrimination. *Conclusions*: ML models using routinely available ED variables improved in-hospital mortality prediction compared with PESI in non-COVID PE, but performance declined in COVID-19 patients, suggesting limited generalizability and the need for COVID-specific refinement and prospective multicenter validation.

## 1. Introduction

With more than 10 million venous thromboembolism events diagnosed worldwide each year, approximately 1 million in the United States and over 700,000 annually across major European countries, pulmonary embolism (PE) represents a substantial and rising global public-health burden [1]. PE is often caused by deep vein thrombosis (DVT). Its severity can vary significantly [2,3] and even asymptomatic PE can produce life-long complications (such as chronic thromboembolic pulmonary hypertension) [4].

Against this background of an already substantial global PE burden, the coronavirus disease 2019 (COVID-19) pandemic has introduced a new and powerful driver of thromboembolic disease, both in the venous and arterial circulation [5]. The infection frequently triggers a profound systemic inflammatory response that predisposes patients to significant thrombotic complications [6]. Alongside the thromboembolic events seen in sepsis, patients often develop a distinct pulmonary micro-thrombotic pattern driven by massive coagulation activation with intense inflammatory and immune responses, termed immunothrombosis [7]. This process can lead to widespread thrombotic microangiopathy and alveolar damage [8]. 

COVID-19 is strongly associated with pulmonary thrombosis and embolism, with PE rates varying by clinical severity—rare in outpatients (0–1.1%), higher in hospitalized cohorts (0.9–8.2%), and highest in Intensive Care Unit (ICU) patients (1.8–18.9%). Autopsy studies further suggest a broader thrombotic picture, reporting PE in 6–23% of deaths alongside widespread in-situ thrombosis and microthrombi [9].

In patients with PE, clinical prognostic tools such as Pulmonary Severity Index (PESI) with its simplified version (sPESI), BOVA, FAST and European Society of Cardiology risk score (ESC), alongside biomarkers and imaging of right ventricular dysfunction, have been extensively validated in general PE populations to estimate short term mortality and adverse events. However, when these scores have been applied specifically to patients with acute PE and concomitant COVID-19, their performance has been only modest. In a retrospective cohort of 85 COVID-19 patients with computed tomography (CT)-confirmed PE, Rodrigues et al. [10] reported that PESI, sPESI, BOVA, FAST and the ESC risk stratification scheme showed limited discrimination for 7- and 30-day mortality (AUCs 0.60–0.73 and 0.54–0.64, respectively), with PESI only slightly outperforming the others. These findings suggest that prognostic tools derived in pre-COVID PE populations may underestimate risk in this setting and highlight the need for more refined prediction strategies.

Machine learning (ML) models were intensely studied in the recent literature as adjuvants for a more accurate prediction of PE patients’ prognosis. Eini et al. [11], in a meta-analysis encompassing 17 studies on 844,071 cases concluded that ML models (out of which Logistic Regression (LR), Random Forests (RF), Support Vector Machines (SVM), XGBoost and Neural Networks were the most commonly used) tend to outperform traditional stratification tools (namely PESI) in PE mortality prediction. Few of those models take into account severe acute respiratory syndrome—coronavirus-2 (SARS-CoV-2) related predictors. Mesinovic et al. [12] published a study on a large cohort (800,000 patients) in which they tried to predict the incidence of PE in patients with COVID-19, utilizing demographics (age, sex, region of admission), comorbidities and symptoms, highlighting age, overall presence of symptoms, shortness of breath and hypertension as key PE predictors. In another study, conducted by Teodoru et al. [13], machine learning algorithms, integrated in a clinical decisional algorithm, were used to assess the role of the neutrophil-to-lymphocyte ratio (NLR) in determining the in-hospital mortality risk in patients with PE. The authors concluded that higher NLR is associated with higher in-hospital mortality.

The primary objective of this study is to test a machine learning model capable of accurately predicting in-hospital mortality among patients presenting to the emergency department with acute pulmonary embolism using clinical, imaging and laboratory findings available in the emergency department, and to assess its transportability regardless of their SARS-CoV-2 infection status.

## 2. Materials and Methods

### 2.1. Study Design

**Figure 1:**
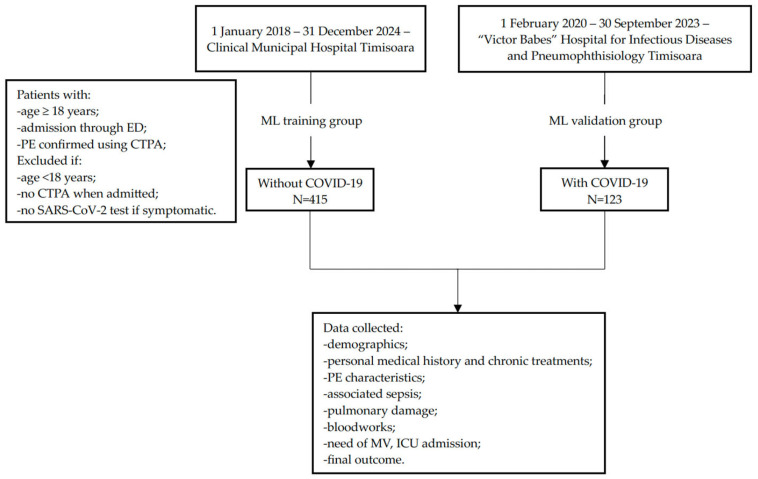
Study flow diagram.

A retrospective, observational cross-sectional study was undertaken using electronic medical records. Data extracted from the Timișoara Clinical Municipal Hospital for the period 1 January 2018–31 December 2024 was used as the ML training group (N = 415). For validation, already collected data from the “Victor Babeș” Hospital for Infectious Diseases and Pneumophthisiology (N = 123), Timișoara, for the period 1 February 2020–30 September 2023 was used [3]. The study flowchart is depicted in Figure 1.

### 2.2. Data Collection

Patients were identified via keyword-based queries of diagnostic codes, specifically ICD-10, I26.0 and I26.9. A subsequent search of free-text diagnosis fields was performed using targeted keywords. All identified cases were reviewed for completeness of required variables, after which pre-specified inclusion and exclusion criteria were applied.

All the required data had been collected utilizing Microsoft Excel 2021, Microsoft Corporation, Redmond, WA, USA. The final database was composed of data regarding: demographics (sex, age, date of admission, residence), PE data (location of the thrombus, PESI class and score (computed during ED stay), Wells rule, Padua score, IMPROVE-VTE score, chosen treatment—thrombolysis, anticoagulant of choice), personal medical history, chronic treatments (antiplatelet drugs/anticoagulants), associated sepsis or other infections, necessity of mechanical ventilation, ICU admission, final outcome and ED laboratory analysis. Sepsis or suspected sepsis reflects ED-time diagnosis; the ED suspicion was subsequently confirmed during the patient’s in-hospital course. Sepsis developed during the hospital admission was not considered. ICU admission was defined as any ICU stay occurring at any point during the hospitalization, including cases where the patient was transferred to the ICU immediately after hospital admission from ED. IMV and NIV need were recorded during the time of hospital stay (including initiation in the ED when applicable). IMPROVE-VTE and Padua scores, associated infections, anticoagulant of choice, and GGT and cholesterol levels were not available during patients’ ED stay, only during hospitalization.

Both during the pandemic and in the pre- and post-pandemic periods, the predictors included only ED-colected data: CT scans interpreted by the on-call radiologist during the ED stay, laboratory tests obtained in the ED prior to admission, as well as all other variables included in the prediction model which were extracted from the patient’s medical history, clinical/paraclinical assessment (e.g., suspected sepsis, obesity). The PESI score was calculated before in hospital admission using the information available at that time.

### 2.3. Inclusion and Exclusion Criteria

Inclusion criteria were: age ≥18 years; admission through the ED; PE confirmed by chest CT pulmonary angiography (CTPA). For patients with suspected SARS-CoV-2 infection, confirmation by reverse-transcription polymerase chain reaction (RT-PCR) (or rapid test) was additionally required. Before March 2020, all patients diagnosed with pulmonary embolism in the ED and admitted to the Emergency Municipal Hospital from Timișoara were included in the study. Starting in March 2020, with the onset of the pandemic, SARS-CoV-2 PCR testing in the ED prior to admission was mandatory for patients who met the suspected case definition (dyspnea, fever, cough, COVID-19 contact within the previous 14 days, or imaging findings suggestive of SARS-CoV-2 infection). After rapid antigen tests became available, all patients meeting the suspected case definition were tested in the ED, and if the rapid test was negative, suplimentary PCR testing was performed. Non-COVID patients were admitted from the ED to the Internal Medicine ward of Emergency Municipal Hospital, whereas SARS-CoV-2–positive patients were admitted to the “Victor Babeș” Hospital for Infectious Diseases and Pneumophthisiology.

Exclusion criteria were: age < 18 years, lack of CTPA upon admission and lack of SARS-CoV-2 testing (after March 2020).

### 2.4. Statistical Workflow

Data analysis and graphical representation have been done using R v4.5.1, R Foundation for Statistical Computing, Vienna, Austria, packages *broom, dplyr, e1071, forcats, ggplot2, logistf, pROC, purrr, randomForest, SHAPforxgboost, stringr, tibble, tidyr, xgboost*.

Prior to analysis, all data were de-identified and assessed for completeness and consistency. Flagged entries were verified against available metadata.

Normality of the data has been assessed using Shapiro-Wilk test. The descriptive statistics encompass median and interquartile ranges for numeric variables, while categorical variables are described using absolute values and percentages. Basic comparisons were done using non-parametric tests (Chi square or Monte Carlo simulation with 10,000 samples for categorical variables and Mann Whitney U for the continuous ones).

For the parameters included in the further analysis, univariate models were used to explore their potential as predictors of the outcome. The coefficient ± standard error (B ± SE), odds ratios (OR) and 95% confidence intervals (95% CI) are given. The outcome used for all the models is in-hospital mortality.

Three machine learning algorithms (RF, XGBoost, and SVM) were trained and internally validated on the non-COVID cohort (80/20) using the following ED-available variables: PESI class, NT-proBNP, D-dimer, neutrophils, platelets, sepsis suspicion, site of PE, obesity, urban residence and heart failure grade. Afterwards, the same three models were externally validated on the entire COVID-19 cohort. The models were evaluated using sensitivity, specificity, ROC-AUC, Brier Score and Youden J. Where given, 95% CI were computed using DeLong’s method for the AUC and exact binomial intervals for sensitivity and specificity. RF models were trained using 1000 trees, with the number of variables randomly sampled each split set to the square root of the number of predictors. XGBoost models were trained using a binary logistic objective function, with a priori fixed hyperparameters to reduce overfitting (maximum tree depth = 3, learning rate = 0.05, subsample = 0.8, column subsampling rate = 0.8). For the SVM models, a radial basis function kernel was used. Feature normalization consisted of predictors standardization to zero mean and unit variance. Initial hyperparameters were: cost = 1 and kernel width = 1/p. Class imbalance for RF and SVM was handled using inverse-frequency class weights computed from the training data set, while for XGBoost scale_pos_weight parameter, calculated from the ratio of negative to positive outcomes in the training set.

To assess robustness of internal performance estimates and reduce dependence on a single random split, we additionally performed repeated stratified k-fold cross-validation within the non-COVID development cohort (k = 5 folds, repeated 20 times)

Calibration in external validation was assessed using calibration-in-the-large (intercept), calibration slope, and Brier score based on logistic recalibration of outcomes on the logit of predicted probabilities. As a sensitivity analysis, recalibration (intercept-only and intercept + slope) was applied to quantify and correct systematic miscalibration in the COVID-19 cohort.

The reported probability values were two-tailed. *p*-values below 0.05 were considered statistically significant, while values below 0.1, marginally significant.

### 2.5. Ethics

The study was approved by the Ethics Committees of the Clinical Municipal Hospital (No. E-46721/11.11.2025) and of the “Victor Babes” University of Medicine and Pharmacy Timisoara (No. 19/2020). All data were anonymized prior to conducting the analysis. The external validation dataset was originally collected under the approval of the Ethics Committee No. 9148/3 October 2023 of “Victor Babes” Hospital for Infectious Disease and Pneumophtisiology from Timisoara and the data was shared in de-identified form. Considering the retrospective nature of the study, the patient’s informed consent was waived.

## 3. Results

All the numeric variables had non-normal distribution.

A total of 538 patients were included in the final analysis. Of these, 439 patients (81.59%) survived and 99 (18.41%) died during admission. Mortality was higher in the COVID-19 population (30.08%, 37 of 123 patients) compared with the non-COVID population (14.93%, 62 of 415 patients). Baseline demographic, clinical, and laboratory characteristics comparing survivors and non-survivors are presented in Table 1. The median age of the cohort was 70 years (IQR 61–78) and did not differ significantly between survivors and non-survivors (70.5 vs. 70 years, *p* = 0.242). Male patients accounted for 49.9% of the cohort, with no significant difference across outcome groups (51.3% vs. 42.5%, *p* = 0.202). Non-survivors were more commonly classified in higher-risk categories, especially PESI class V (45.1% vs. 26.8%), while survivors more often fell into lower-risk strata. The vascular distribution of emboli was predominantly central. Need for ventilatory support was strongly associated with mortality. Invasive mechanical ventilation was required in 53.4% of non-survivors compared with 10.6% of survivors (*p* < 0.001). Non-invasive ventilation was also far more frequent among non-survivors (51.0% vs. 2.3%, *p* < 0.001).

The COVID-19 cohort had near-complete availability of model predictors (mean missing predictors per patients 0.056 with a median of 0). Neutrophil counts had a standardized mean difference of 0.041, with a population stability index of 0.09. All the other numeric variables had satisfactory distributional shifts. Categorical case-mix differed for heart failure class and obesity (*p* < 0.01). Furthermore, thrombus localization differed between cohorts (*p* < 0.001). COVID-19 patients had higher odds of distal thrombus localization (segmental or smaller) compared with proximal localization (trunk/main/lobar) than patients without COVID-19 (OR 5.51, 95% CI 3.26–9.5, *p* < 0.001).

The univariate analysis is presented in Table 2. PESI class 5, NT-proBNP, neutrophil count, platelet count, sepsis, obesity, NYHA IV heart failure, urban residence and pulmonary hypertension were significant predictors.

In the internal validation cohort, all machine-learning models demonstrated good calibration and discrimination, with Brier scores ranging from 0.106 to 0.139. The Random Forest model achieved the lowest Brier score (0.106), indicating the best overall calibration, while XGBoost and SVM showed Brier scores of 0.139 and 0.122, respectively. In terms of discrimination, both XGBoost and Random Forest achieved high sensitivity (0.889), with XGBoost providing a balanced specificity of 0.800 and the highest Youden index (0.689). The SVM model showed slightly lower sensitivity (0.778), but comparable specificity (0.800), resulting in a Youden index of 0.578. In repeated stratified 5-fold cross-validation of the non-COVID cohort (20 repeats), mean AUC ± SD was 0.728 ± 0.197 for SVM, 0.867 ± 0.103 for XGBoost, 0.846 ± 0.108 for random forest, and 0.663 ± 0.143 for PESI class.

**Figure 2:**
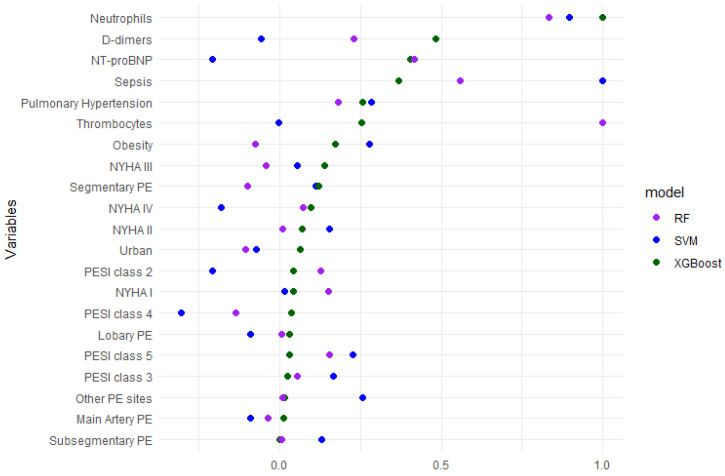
Global variable importance of the predictors used for the models (predictors are ordered according to their importance ranking in the XGBoost model).

For external validation on the COVID-19 cohort, XGBoost maintained the most balanced performance, with a sensitivity of 0.629 (95% CI 0.449–0.785), specificity of 0.647 (95% CI 0.536–0.748), and a Youden index of 0.276, alongside a Brier score of 0.217, an intercept of −0.543 and a slope of 0.214. Following intercept-and-slope recalibration, the Brier score improved to 0.202. Random Forest showed high specificity (0.847, 95% CI 0.753–0.916)) but markedly lower sensitivity (0.429, 95% CI 0.263–0.606), yielding the same Youden index (0.276), a higher Brier score (0.245), an intercept of 0.647 and a slope of 0.588. After correcting the calibration, the Brier score improved to 0.197. The SVM model failed to demonstrate discriminative ability in the external validation, with sensitivity and specificity not estimable and a Brier score of 0.215, indicating limited clinical utility in this setting. The global variable importance of predictors is presented in Figure 2.

**Figure 3:**
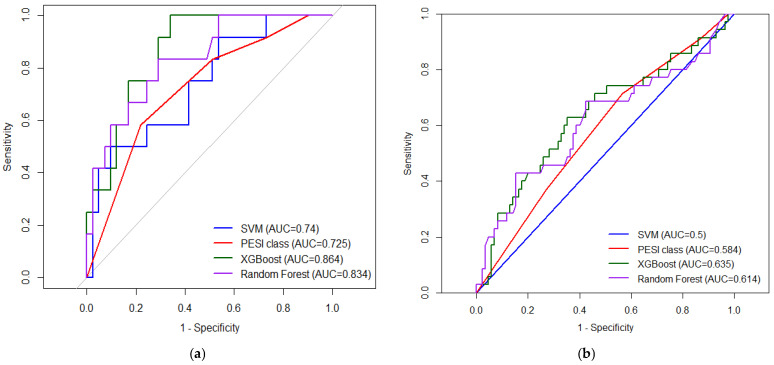
ROC and AUC for PESI and ML models used (SVM, XGBoost and RF) in the training cohort (**a**) compared to the validation cohort (**b**).

ROC analysis assessing prediction of mortality demonstrated that machine-learning models outperformed PESI class alone. XGBoost showed the best discrimination (AUC = 0.864, 95% CI 0.726–0.992), followed by Random Forest (AUC = 0.834, 95% CI 0.723–0.993) and SVM (AUC = 0.740, 95% CI 0.686–0.839). PESI class had modest predictive value (AUC = 0.725, 95% CI 0.552–0.825), lower than both tree-based ML models (Figure 3). SVM models had zero discrimination on the COVID-19 validation set, while XGBoost had an AUC of 0.635 (95% CI 0.520–0.751), in comparison to 0.614 (95% CI 0.495–0.734) for RF and 0.584 (95% CI 0.477–0.691) for the PESI score.

In the external COVID-19 cohort, applying the development-derived triage threshold (targeting above 90% sensitivity in the ML development cohort) resulted in no patients exceeding the cutoff, under the observed case-mix shift. Following cohort-specific recalibration, sensitivity increased to 0.914 (95% CI 0.769–0.982) with a specificity of 0.106 (95% CI 0.05–0.192) and negative predictive value 0.75 (95% CI 0.428–0.945). Ten percent of the patients were classified as low risk, the model missing 3 deaths.

## 4. Discussion

In this retrospective cohort of 538 patients admitted through the emergency department with confirmed PE with or without COVID-19, in-hospital mortality was 18.4%. Non-survivors more frequently presented with higher PESI classes, sepsis, and a need for ventilatory support, whereas age and sex distributions were similar between outcome groups. The tree-based machine-learning models, particularly XGBoost and Random Forest, showed better discrimination for in-hospital mortality than PESI class alone (AUC 0.864 and 0.834 vs. 0.725), while SVM performance was comparable to that of established clinical scores.

The superior discrimination of XGBoost and Random Forest over PESI class aligns with previous work showing that ML-based models generally outperform traditional clinical scores for PE mortality prediction. In the meta-analysis by Eini et al., ML algorithms, including tree-based models and logistic regression, consistently showed higher AUCs than PESI in diverse PE populations [11]. Our findings extend these observations to an emergency department cohort and reinforce the notion that non-linear interactions between clinical and laboratory variables are relevant for risk stratification but are not captured by conventional scoring systems. Developing these types of tools, capable of helping physicians with early risk stratification is utterly useful especially in nowadays overcrowded emergency departments.

The study is particularly relevant in the context of COVID-19, a condition strongly associated with pulmonary thrombosis and embolism through mechanisms of immunothrombosis and microvascular injury [14]. Previous studies have shown that PESI, sPESI, BOVA and ESC risk stratification have only modest performance in patients with concurrent COVID-19 and PE [10]. By developing and validating models primarily in a non-COVID cohort and then applying them to patients with SARS-CoV-2 infection, our approach explores whether a single, unified risk-stratification framework can be used across these clinically distinct but pathophysiologically related entities. Depending on the final performance in the COVID-19 subgroup, this may support either the use of shared prediction tools or the need for COVID-specific recalibration.

According to previous validation studies, higher categories of the PESI identify patients at markedly increased short-term risk, including those with COVID-19–related pulmonary embolism [5,15]. We noted similar patterns, with PESI class V being associated with more than a five-fold increase in the odds of in-hospital mortality. Still, authors recommend to not use PESI in patients with PE secondary to COVID-19 until recalibrated accordingly. In this context, the higher PESI, Padua, and IMPROVE-VTE scores observed among non-survivors further emphasize the cumulative contribution of comorbidities and baseline thrombotic risk to short-term adverse outcomes.

In agreement with earlier studies that indicated elevated cardiac strain biomarkers are associated with increased risk of poor in-hospital outcomes and death in PE patients [16,17], NT-proBNP levels in our cohort were notably connected to mortality, reinforcing its function as a surrogate indicator of right ventricular dysfunction and hemodynamic stress. Higher NT-proBNP values were associated with a higher risk of in-hospital death in univariate analysis.

In the current study, higher neutrophil counts were linked to increased mortality, aligning with evidence that shows elevated inflammatory marker, especially the neutrophil-to-lymphocyte ratio, can predict both in-hospital and long-term mortality in patients with acute PE [18,19,20]. Neutrophilia, in patients with COVID-19, may be a sign of NETosis, a process in which neutrophils release neutrophil extracellular traps (NETs)—web-like DNA structures coated with enzymes such as myeloperoxidase and neutrophil elastase [21,22]. These NETs can promote clot formation by providing a sticky surface that helps platelets and fibrin build up. In several VTE/PE studies, markers linked to NET formation have been found at higher levels, which supports the idea that excessive NETs may contribute to more severe PE and worse outcomes [23]. This mechanism may be especially important in infection-related inflammation, including COVID-19, where immunothrombosis has been described. Nonetheless, inflammation seems to play a significant role in PE development and the importance of inflammatory markers in predicting the outcome of patients with PE has been probed in other papers, but still requires further studying.

The strong connection between sepsis and mortality found in our group mirrors what registry and population-based studies indicate: that acute PE in septic patients comes with significantly higher in-hospital mortality, extended hospital stays, and greater healthcare resource use [24]. This interaction is likely due to the combined effects of inflammatory dysregulation, endothelial injury, and coagulation abnormalities [25].

On the other hand, the seemingly protective association observed for obesity and chronic cardiopulmonary conditions aligns with the “obesity paradox” described in the literature on pulmonary embolism and venous thromboembolism, in which higher body mass index has been linked to lower short-term mortality following acute PE [26]. In our cohort, obesity and pre-existing pulmonary hypertension were more prevalent among survivors, as patients with chronic cardiopulmonary disease may present earlier or receive closer monitoring and more intensive anticoagulant therapy. Alternatively, this pattern may be explained by greater metabolic reserve, altered inflammatory responses, or earlier clinical recognition in patients with established comorbidities.

From a clinical perspective, our findings support the potential role of ML-based tools as decision aids in the initial risk assessment of patients presenting with suspected or confirmed PE. Overall, XGBoost demonstrated the most favorable balance between sensitivity and specificity in the internal validation setting. In external validation using the COVID-19 cohort, discrimination and calibration deteriorated across algorithms, consistent with clinically meaningful dataset shift between cohorts, including a higher baseline mortality rate, differing comorbidity and changes in inflammatory profiles and thrombus localization. Notably, COVID-19 patients exhibited a distal shift in thrombus location, with substantially higher odds of segmental/subsegmental involvement compared with proximal involvement than non-COVID patients (OR 5.51, 95% CI 3.26–9.50; *p* < 0.001). These findings suggest that while ML models may retain value as risk stratification aids, transport to new clinical contexts requires recalibration and explicitly defined operating points aligned with intended use.

This study has several limitations. First, its retrospective design based on electronic medical records makes it susceptible to selection bias, misclassification and missing data. Second, although the overall cohort size was moderate, the number of deaths was relatively limited, which increases the risk of overfitting and constrains the complexity of the models that can be reliably trained. Third, all patients were recruited from two hospitals in a single region, which may limit generalizability to other healthcare systems and populations with different baseline risk profiles or treatment strategies.

Despite these limitations, the use and further in-depth study of these prediction tools could be extremely useful in the ED, particularly for determining the appropriate level of monitoring—on the ward, in an intermediate care unit, or in the ICU—according to short-term mortality risk, as admission decisions there are made under significant time constraints and heightened stress. Additionally, important strengths include the use of systematically collected ED data, inclusion of both clinical and laboratory variables readily available at presentation, and the attempt to externally validate models in an independent cohort of patients with COVID-19.

## 5. Conclusions

Our findings suggest that in patients with acute PE, especially those with concomitant COVID-19, traditional risk scores (including PESI) show reduced prognostic performance. Machine-learning models built from routinely available clinical and laboratory data showed better mortality discrimination in non-COVID PE, but their performance declined in the COVID-19 subgroup, indicating the need for disease-specific refinement.

Further prospective, multicenter studies are warranted to develop and validate more accurate risk-assessment tools to support early clinical decision-making in this population.

## Figures and Tables

**Table 1:** Descriptive statistics of the whole patient population.

Variable	All Patients (N = 538)	Survivors (N = 439)	Non-Survivors (N = 99)	*p*-Value *^,^**
Age (years) ^(a)^	70 (61–79)	69 (61–79)	73 (65–83)	0.242
Gender (Male) ^(b)^	264 (49.2%)	222 (50.7%)	42 (42.4%)	0.085
Urban residence ^(b)^	314 (58.5%)	272 (62.1%)	42 (42.4%)	<0.001 **
PESI class 2 ^(b)^	64 (14%)	60 (15.6%)	4 (5.6%)	0.004 *
PESI class 3 ^(b)^	113 (24.8%)	98 (25.5%)	15 (21.1%)	
PESI class 4 ^(b)^	105 (23%)	87 (22.6%)	18 (25.4%)	
PESI class 5 ^(b)^	135 (29.6%)	103 (26.8%)	32 (45.1%)	
PESI score ^(a)^	105 (83–131)	103 (80–128)	133 (103–170)	<0.001 **
IMPROVE-VTE score ^(a)^	1 (1–2)	1 (1–2)	2 (1–3)	<0.001 **
Padova score ^(a)^	3 (2–4)	3 (2–4)	3.5 (3–6)	<0.001 **
Thrombus localisation				
Trunk ^(b)^	18 (3.4%)	15 (3.4%)	3 (3%)	0.067
Main ^(b)^	128 (23.8%)	106 (24.2%)	22 (22.2%)	
Lobar ^(b)^	140 (26.1%)	116 (26.5%)	24 (24.2%)	
Segmentary ^(b)^	146 (27.2%)	110 (25.1%)	36 (36.4%)	
Subsegmental ^(b)^	17 (3.2%)	15 (3.4%)	2 (2%)	
Other ^(b)^	88 (16.4%)	76 (17.4%)	12 (12.2%)	
Comorbidities				
Heart failure NYHA I ^(b)^	62 (11.5%)	44 (10%)	18 (18.2%)	0.002 *
Heart failure NYHA II ^(b)^	195 (36.3%)	173 (39.5%)	22 (22.2%)	
Heart failure NYHA III ^(b)^	84 (15.6%)	62 (14.2%)	22 (22.2%)	
Heart failure NYHA IV ^(b)^	13 (2.4%)	7 (1.6%)	6 (6.1%)	
Pulmonary hypertension ^(b)^	234 (43.6%)	210 (47.9%)	24 (24.2%)	<0.001 **
Type 2 DM ^(b)^	105 (20.6%)	89 (21.6%)	16 (16.3%)	0.153
Cancer ^(b)^	98 (19.1%)	79 (19.1%)	19 (19.4%)	0.522
Obesity ^(b)^	168 (31.3%)	152 (34.7%)	16 (16.2%)	0.004 **
Sepsis ^(b)^	75 (14%)	29 (6.6%)	46 (46.5%)	<0.001 **
Associated infections ^(b)^	162 (37.7%)	138 (37.7%)	24 (37.5%)	0.975
Anticoagulant of choice				
Nadroparinum ^(b)^	57 (10.8%)	41 (9.6%)	16 (16.2%)	0.071
Unfractionated heparin ^(b)^	49 (9.6%)	29 (7%)	20 (20.4%)	0.001 **
Enoxaparine ^(b)^	185 (36.1%)	146 (35.2%)	39 (39.8%)	0.229
Antivitamin K ^(b)^	18 (4.6%)	18 (5.5%)	-	0.089
Fondaparinux ^(b)^	299 (78.1%)	273 (84.8%)	26 (42.6%)	<0.001 **
Laboratory results				
Leucocytes [×10^3^/μL] ^(a)^	9.8 (7.17–12.22)	9.55 (7.03–11.89)	11.66 (7.44–17.54)	<0.001 **
Neutrophils [×10^3^/μL] ^(a)^	7.8 (5.77–10.3)	7.35 (5.70–9.60)	10.12 (6.00–15.70)	<0.001 **
Limphocytes [×10^3^/μL] ^(a)^	1.1 (0.74–1.66)	1.15 (0.81–1.90)	0.84 (0.48–1.00)	<0.001 **
Thrombocytes [×10^3^/μL] ^(a)^	228 (160–293)	230 (168–295)	200 (120–280)	0.105
Creatinin [mg/dL] ^(a)^	1 (0.79–1.31)	1.00 (0.80–1.30)	0.90 (0.71–1.35)	0.786
Total bilirubin [mg/dL] ^(a)^	0.56 (0.4–0.8)	0.53 (0.40–0.80)	0.60 (0.40–0.90)	0.134
GOT [U/L] ^(a)^	32 (23–59.35)	31 (22–56)	40 (30–64)	<0.001 **
GPT [U/L] ^(a)^	36.4 (23–70.5)	33 (22–67)	55 (31–79)	0.016 *
GGT [U/L] ^(a)^	55 (32–94)	54.50 (31–89)	78 (50–118)	0.001 **
Glicemia [mg/dL] ^(a)^	125 (105–163.5)	122 (101–163)	139 (120–172)	0.210
Sodium [mEq/L] ^(a)^	137 (134–140)	137 (134–140)	137 (135–140)	0.453
Potassium [mEq/L] ^(a)^	4.1 (3.85–4.4)	4.10 (3.80–4.40)	4.10 (3.90–4.47)	0.367
Procalcitonin [ng/mL] ^(a)^	0.3 (0.06–0.44)	0.23 (0.05–0.40)	0.44 (0.30–2.55)	<0.001 **
Cholesterol [mg/dL] ^(a)^	175 (144–224)	168 (144–200)	245 (199–273)	<0.001 **
INR ^(a)^	1.1 (1–1.21)	1.12 (1.00–1.20)	1.15 (1.04–1.30)	<0.001 **
APTT [s] ^(a)^	23.7 (21.5–26.35)	23.90 (21.80–26.50)	22.70 (20.90–25.66)	0.382
PT [s] ^(a)^	13.3 (12.4–14.5)	13.30 (12.30–14.20)	13.60 (12.75–15.45)	<0.001 **
D-dimers [µg/mL] ^(a)^	4.5 (2.3–8.37)	4.50 (2.30–8.55)	3.76 (2.10–6.65)	0.927
CK-MB [ng/mL] ^(a)^	25 (16–43.5)	24.50 (15.00–39.00)	34 (22–56)	0.004 **
NT-proBNP [pg/mL] ^(a)^	391 (139–2258)	393 (125–1822)	388 (177–5848)	0.063
IMV ^(b)^	89 (16.7%)	42 (9.7%)	47 (47.5%)	<0.001 **
NIV ^(b)^	29 (13.1%)	4 (2.3%)	25 (51%)	<0.001 **

^(a)^ Median (IQR), Mann Whitney U test. ^(b)^ Absolute value (%), either Chi-Square statistical test or Monte Carlo Simulation with 10,000 samples. *^,^** statistically significant. Abbreviations: APTT—Activated Partial Thromboplastin Time, CK-MB—Creatine Kinase—MB, DM—Diabetes Mellitus, GGT—Gamma-glutamyl Transferase, GOT—Glutamic-oxaloacetic transaminase, GPT—Glutamic-pyruvic transaminase, IMPROVE-VTE—IMPROVE risk Score for Venous Thromboembolism, IMV—invasive mechanical ventilation, INR—International normalized ratio, NIV—non-invasive mechanical ventilation, NYHA—New York Heart Association, PESI—Pulmonary Severity Index, PT—prothrombin time.

**Table 2:** Univariate analysis of the predictors.

Variable		B ± SE	OR	95% CI for OR	*p*-Value
PESI class	2	−0.243 ± 0.789	0.78	0.16–4.15	0.758
	3	1 ± 0.645	2.72	0.88–11.95	0.121
	4	1.124 ± 0.646	3.08	0.99–13.55	0.082
	5	1.715 ± 0.624	5.56	1.89–23.78	0.006 **
NT-proBNP ^(a)^		0.185 ± 0.079	1.20	1.03–1.41	0.018 *
D-dimer ^(a)^		0.066 ± 0.141	1.07	0.81–1.4	0.640
Neutrophils ^(a)^		0.173 ± 0.036	1.19	1.11–1.28	<0.001 **
Platelets ^(a)^		0.11 ± 0.036	1.12	1.04–1.2	0.002 **
Sepsis		2.458 ± 0.279	11.68	6.81–20.41	<0.001 **
Central artery involvement		0.037 ± 0.674	1.04	0.31–4.75	0.956
Pulmonary hypertension		−1.136 ± 0.254	0.32	0.19–0.52	<0.001 **
Obesity		−1.11 ± 0.292	0.33	0.18–0.57	<0.001 **
Urban residence		−0.799 ± 0.226	0.45	0.29–0.7	<0.001 **
Heart failure NYHA IV		−0.739 ± 0.252	0.48	0.29–0.78	0.003 **

^(a)^ Entered into the model in log scale. For log-transformed variables, ORs correspond to a 1-unit increase on the scale. *^,^** statistically significance, *p* < 0.05, *p* < 0.01, Abbreviations: B ± SE, coefficient ± standard error; OR, odds ratios; 95% CI, 95% confidence intervals.

## Data Availability

The data presented in this study are available on request from the corresponding author.

## Abbreviations

The following abbreviations are used in this manuscript:

95% CI	95% confidence intervals
APTT	Activated Partial Thromboplastin time
AUC	Area under the curve
B ± SE	exponent ± standard deviation
CI	Confidence interval
CK-MB	Creatinkinase-MB
COVID-19	Coronavirus disease–19
CT	computed tomography
DM	Diabetes mellitus
DVT	Deep vein thrombosis
ED	Emergency Department
GGT	Gamma-glutamyl Transferase
GOT	Glutamic-oxaloacetic transaminase
GPT	Glutamic-pyruvic transaminase
ICD	International Classification of Diseases
ICU	Intensive Care Unit
IMV	Invasive mechanical ventilation
INR	International normalized ratio
LR	Logistic regression
NIV	Noninvasive mechanical ventilation
NYHA	New York Heart Association
OR	Odds ratio
PESI	Pulmonary Severity Index
PT	Prothrombin Time
RF	Random Forest
ROC	Receiver Operating Characteristic
SARS-CoV-2	severe acute respiratory syndrome—coronavirus-2
SVM	Support Vector Machines
